# The transotic approach for vestibular schwannoma: indications and results

**DOI:** 10.1007/s00405-017-4627-3

**Published:** 2017-06-06

**Authors:** Yin Xia, Wenyang Zhang, Yi Li, Xiaobo Ma, Qiang Liu, Jinghua Shi

**Affiliations:** 1 Department of Otorhinolaryngology, Beijing Tiantan Hospital, Capital Medical University, Beijing, 100050 China; 2 Department of Otorhinolaryngology, Peking Union Medical College Hospital, Beijing, 100050 China; 3 Department of Otorhinolaryngology Head and Neck Surgery, Beijing Tongren Hospital, Beijing, 100730 China

**Keywords:** Transotic approach, Vestibular schwannoma, Facial nerve preservation, Cerebrospinal fluid leak

## Abstract

To analyze retrospectively the indications and the results obtained with the transotic approach in a series of patients with vestibular schwannoma. The study included 36 patients from 2007 to 2013 with a vestibular schwannoma that was removed with a transotic approach. All patients underwent preoperative pure tone audiometry, evaluation of facial function and CT and MR imaging. All patients having (1) a hearing loss of more than 50 dB, (2) an average tumor size of 2.7 cm (range 0.5–5.0 cm) and (3) signs of a contracted mastoid (high jugular bulb, anteriorly located sigmoid sinus, low middle cranial fossa, or reduced pneumatization) were selected. The tumor was totally removed in 34 and near-totally removed in 2 patients. The facial nerve was preserved in all patients. The postoperative facial function after 6 weeks was House–Brackmann grade I in 7, grade II in 27, and grade III in 2 patients. All patients presented postoperatively with unilateral total deafness. Seven patients experienced transitory postoperative imbalance. There were two patients who required revision surgery, one with intracranial hemorrhage and another with a CSF leak. There were no deaths and no severe complications such as hemiplegia or intracranial infections. The transotic approach has proven to be of value for the removal of vestibular schwannomas up to 5.0 cm in the presence of temporal bone contraction. Hearing was not preserved; however, other clinical outcomes were very favorable, including high rates of total tumor removal and facial nerve preservation, and low rates of complications.

## Introduction

Vestibular schwannoma is the most common tumor of the cerebellopontine angle. Its treatment options include observation (wait-and-scan policy), radiation treatment and surgery [1]. The most commonly used surgical approaches are the translabyrinthine, retrosigmoid and middle cranial fossa approaches. The appropriate approach should be selected for the individual patient according to tumor size and location with the intent of radical tumor removal, preservation of the facial nerve and, if possible, preservation of the patient’s cochlear function [2]. In the 1960s, William F. House developed the translabyrinthine approach [3], which became very popular because it reduced the mortality of vestibular schwannoma extirpation from 20% (neurosurgical approaches) to 2%. The total loss of hearing was accepted in view of the radical removal of the tumor combined with better preservation of facial function with dramatically reduced postoperative morbidity and mortality [4].

In the 1970s, Ugo Fisch developed the transotic approach [5–7] to avoid three main limitations inherent in the translabyrinthine approach [8]: (1) the reduction of surgical exposure given by the preservation of the middle ear spaces in cases of reduced pneumatization, anterior location of the sigmoid sinus, high jugular bulb and low middle cranial fossa dura; (2) the difficulty in exposing the anterior cerebellopontine angle and, therefore, in separating and preserving under direct vision the intracranial segment of the facial nerve from the anterior pole of the tumor; and (3) the danger of postoperative CSF leakage and meningitis due to the direct contact of the intact middle ear mucosa with the subarachnoid space in the operated mastoid cavity [8]. We introduced the transotic approach in our clinic in 2007, and would like to present a retrospective review of our experience with it.

## Materials and methods

Institutional Review Board (IRB) approval was obtained from our institution. Each patient, or his/her guardian(s), provided written informed consent for the surgical procedures. And all the cases operated by one surgeon.

### Patients

This study consists of a retrospective review of 36 patients (16 males and 20 females) hospitalized from July 2007 to March 2013 who underwent removal of a vestibular schwannoma using the transotic approach. The age of the patients at the time of surgery ranged from 19 to 72 years (mean age 47.2 years), and five patients were above 60 years (Table 1).

**Table 1:** Patients Information and time of surgery

Tumor size (cm)	*N* (%)	Average age of patients	Hearing loss >50 dB	Reduced pneumatization, low middle cranial fossa, anterior sigmoid sinus and high bulb of jugular bulb	Tumor removal		Time of surgery (h)		
					Total	Partial	Exposure	Removal	Total
0.5–2.0	13 (36%)	48	13	13	13	0	2.4	1.5	3.9
2.1–4.0	18 (50%)	45.7	16	18	17	1	2.5	2.1	4.6
4.1–5.0	5 (14%)	50.6	5	5	4	1	3.1	2.8	5.9
Total	36	47.2	34	36	34	2	2.5	2.0	4.5

### Preoperative examinations

1. Hearing levels were assessed with pure tone audiometry using a GSI-61 audiometer. Eleven patients (31%) had a total unilateral deafness, and the hearing loss of other patients averaged 62.7 dB for the four-tone average of 0.5, 1, 2 and 4 kHz (Table 2).

**Table 2:** Clincal outcomes

Tumor size (cm)	*N* (%)	Mean preoperative hearing levels (dB) (0.5–4.0 kHz)	Total unilateral preoperative deafness (number of ears)	Average facial nerve function (HB grade/DEFS)						Vertigo	Intracranial hemorrhage	CSF leak
				Preoperative			Postoperative (6 weeks)					
				I/100%	II/76–99%	III/51–75%	I/100%	II/76–99%	III/51–75%			
0.5–2.0	13 (36%)	65.9	2	13/100%	0	0	6/100%	7/83%	0	1 (2.8%)	0	0
2.1–4.0	18 (50%)	57.3	5	15/100%	3/87%	0	1/100%	17/81%	0	2 (5.6%)	0	0
4.1–5.0	5 (14%)	63.8	4	0	4/82%	1/66%	0	3/77%	2/59%	4 (11.1%)	1 (2.8%)	1 (2.8%)
Total	36	62.7	11	28/100%	7/84%	1/66%	7/100%	27/81%	2/59%	7 (19.5%)	1 (2.8%)	1 (2.8%)

*DEFS* detailed evaluation of facial symmetry
2. The preoperative facial nerve function was assessed in grades according to House–Brackmann and for each HB grade in percentages according to the Fisch detailed examination of facial symmetry (DEFS) [9]. The Fisch DEFS was used to have a quantitative analysis of facial movements in contrast to the HB grading that only assigns these in categories. Table 2 shows that 28 patients (78%) presented preoperatively with a HB grade I (resp. 100% DEFS), 7 patients (19%) with a HB grade II (resp. 84% DEFS) and 1 patient (3%) with a HB grade III (resp. 66% DEFS).
  Examination with the Medtronic KEYPOINT 4 EMG unit measured muscle action potentials by stimulating the facial nerve. A bipolar stimulating electrode was placed at the stylomastoid foramen and the rest recording electrode was placed in the nasolabial groove [10, 11]. The amplitude and latency of compound muscle action potential (CMAP) elicited by the maximum response were recorded and compared between the affected and normal facial nerve. The statistics demonstrated that 28 patients (78%) had normal facial function, 7 patients (19%) mildly abnormal function and 1 patient (3%) moderately abnormal function.
3. Vestibular function: Examined via Ulmer VNG, 3 patients were normal and 33 patients demonstrated a reduced caloric response all on their tumor side.
4. CT scans of the temporal bones were obtained for all patients in the horizontal and coronal planes with a Philips Brilliance 64-slice CT scanner to evaluate the extent of pneumatization of the temporal bones and determine the position of the middle cranial fossa, sigmoid sinus and jugular bulb. All patients had a postoperative CT scan (Fig. 1).

**Fig. 1:**
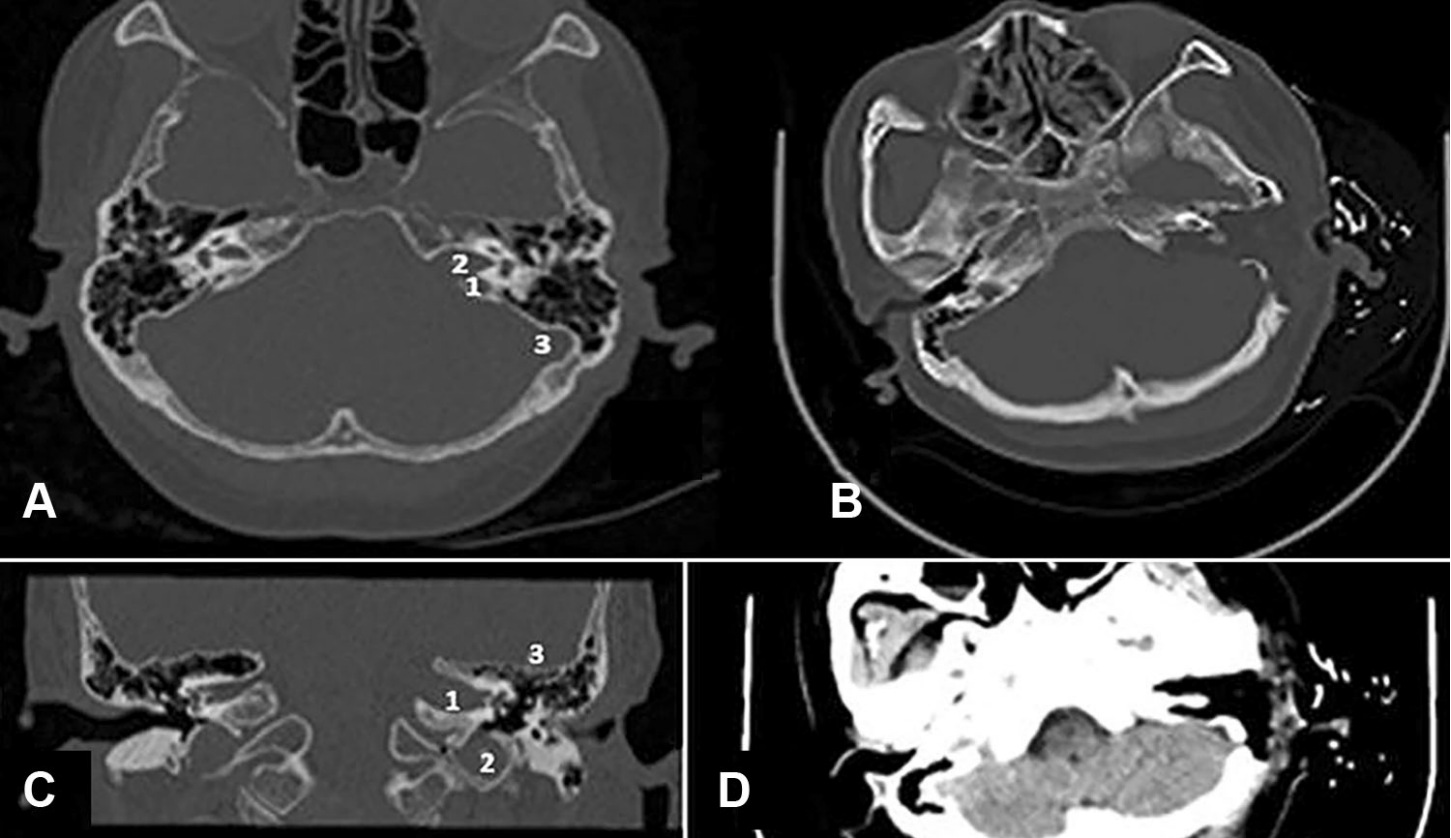
**a** The axial CT shows the high jugular bulb, the enlarged internal auditory canal with the acoustic tumor. *1* high jugular bulb, *2* enlarged internal auditory canal, *3* sigmoid sinus, normal middle cranial fossa dura and pneumatization. **b** The postoperative axial CT demonstrates the obliterated subtotal petrosectomy with radical tumor removal. **c** The coronal HRCT shows the left IAC enlarged by a vestibular schwannoma. *1* enlarged internal auditory canal, *2* high jugular bulb, *3* normal middle cranial fossa dura and pneumatization. **d** The postoperative axial CT of the same patients showing the operative cavity with the radical tumor removal
5. MRI scans of the internal auditory canal cerebellopontine angle and posterior cranial fossa were obtained in all 36 patients via GE Signa 1.5T MRI scanner (T1 weighted post-gadolinium sequences), to determine tumor size. The tumors were 0.5–2 cm in 13 patients (36%), 2–4 cm in 18 (50%) patients and 4–5 cm in 5 patients (14%).

### Inclusion criteria for the transotic approach

Hearing loss of more than 50 dB in all 36 patients, or tumor larger than 2 cm making hearing preservation impossible in 23 patients, and CT scans showing narrow access to the cerebello-pontine angle, such as reduced pneumatization, low middle cranial fossa, anterior sigmoid sinus and/or high jugular bulb (Table 1).

### Surgical technique [5]

General anesthesia and facial nerve monitoring were carried out in all cases. A C-shaped retroauricular incision was made from the temporal region, starting superior to the apex of the pinna, continuing posteriorly and inferiorly to 2 cm below the mastoid tip along the postauricular hair line. An anteriorly based mastoid periosteal flap was developed from the mastoid periosteum and soft tissues. The skin of the external auditory canal was transected at the level of the bony-cartilaginous junction, everted through the external meatus and closed with 4-0 Vicryl sutures. The remaining skin of the external auditory canal and the tympanic membrane with attached malleus handle were removed from the bony external canal. After the initial stages of the mastoidectomy, a subtotal petrosectomy was performed exenterating all pneumatic cell tracts of the middle ear cleft [16]. The fallopian canal was skeletonized from the genu to the stylomastoid foramen. Bone was widely removed to open the infralabyrinthine region and to skeletonize the jugular bulb and the vertical segment of the internal carotid artery. The horizontal segment of the internal carotid artery was followed superiorly to the isthmus of the Eustachian tube, which was exposed. The mucosa of the tympanic ostium of the Eustachian tube was coagulated and the lumen obliterated with bone wax.

**Fig. 2:**
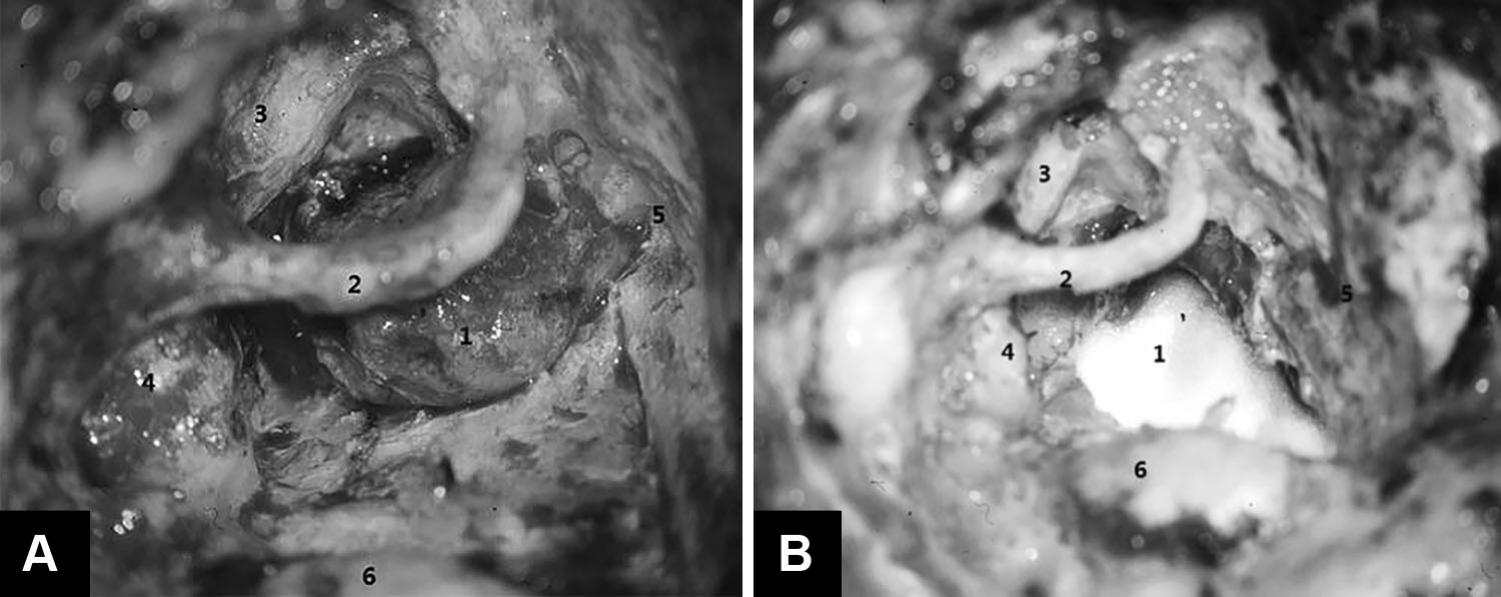
**a** Exposure of vestibular schwannoma by transotic approach (*left ear*). *1* Tumor, *2* skeletonized facial nerve, *3* internal carotid artery, *4* jugular bulb, *5* dura of middle cranial fossa, *6* sigmoid sinus. **b** After tumor removal the posterior fossa dura was reconstructed with temporalis fascia or artificial dura. *1* Artificial dura mater, *2* skeletonized facial nerve, *3* internal carotid artery, *4* jugular bulb, *5* dura of middle cranial fossa, *6* sigmoid sinus

**Fig. 3:**
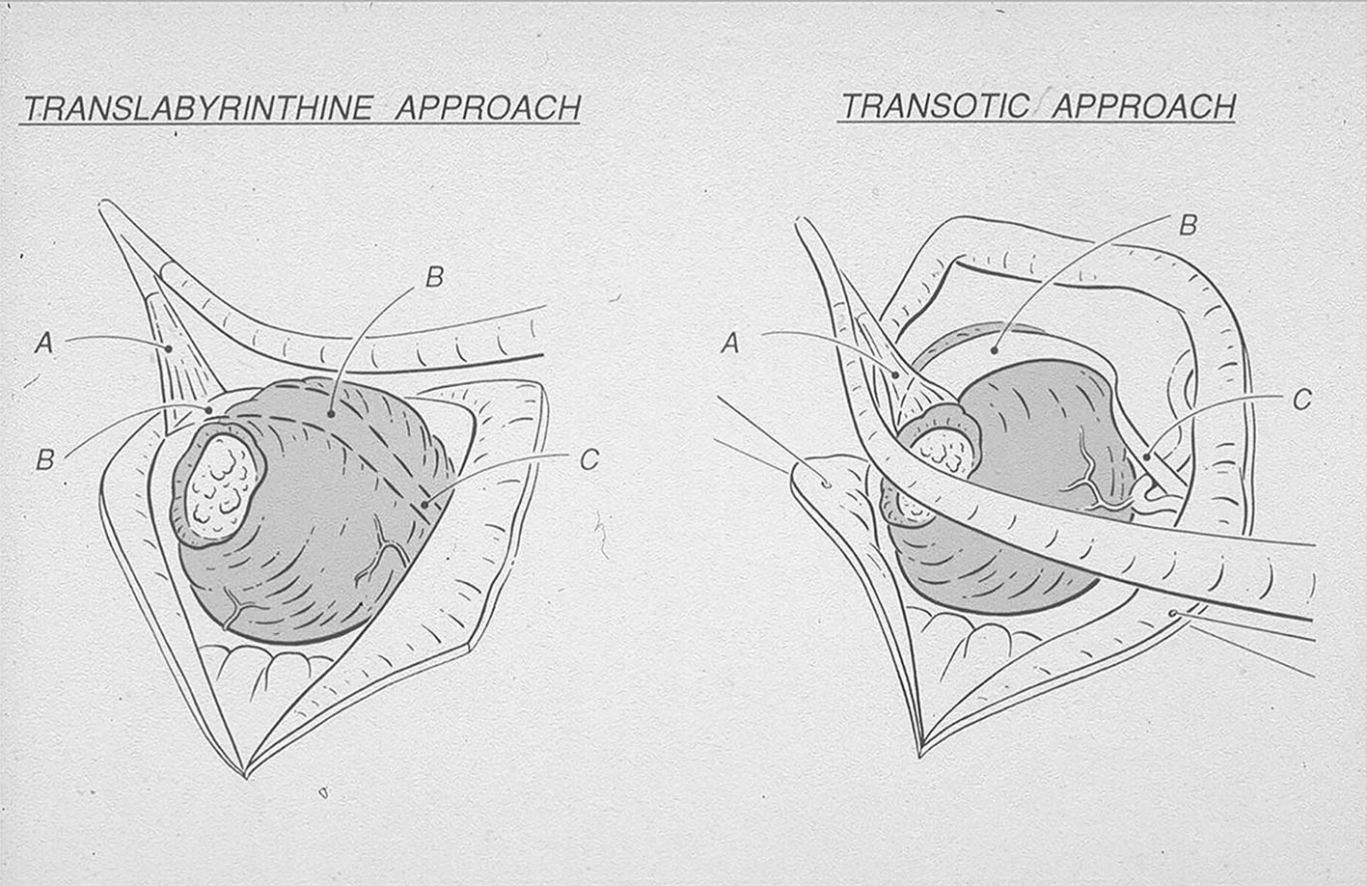
Schematic view showing why the transotic approach (*left side*) improves exposure of the intracranial segment of the facial nerve in the anterior cerebello-pontine angle in respect to the translabyrinthine approach (*right ear*) [5]. **a** Intrameatal segment of facial nerve, **b** intracranial segment of facial nerve, **c** origin of facial nerve from the brainstem (note proximity of anterior inferior cerebellar artery). Courtesy Prof. U. Fisch

**Fig. 4:**
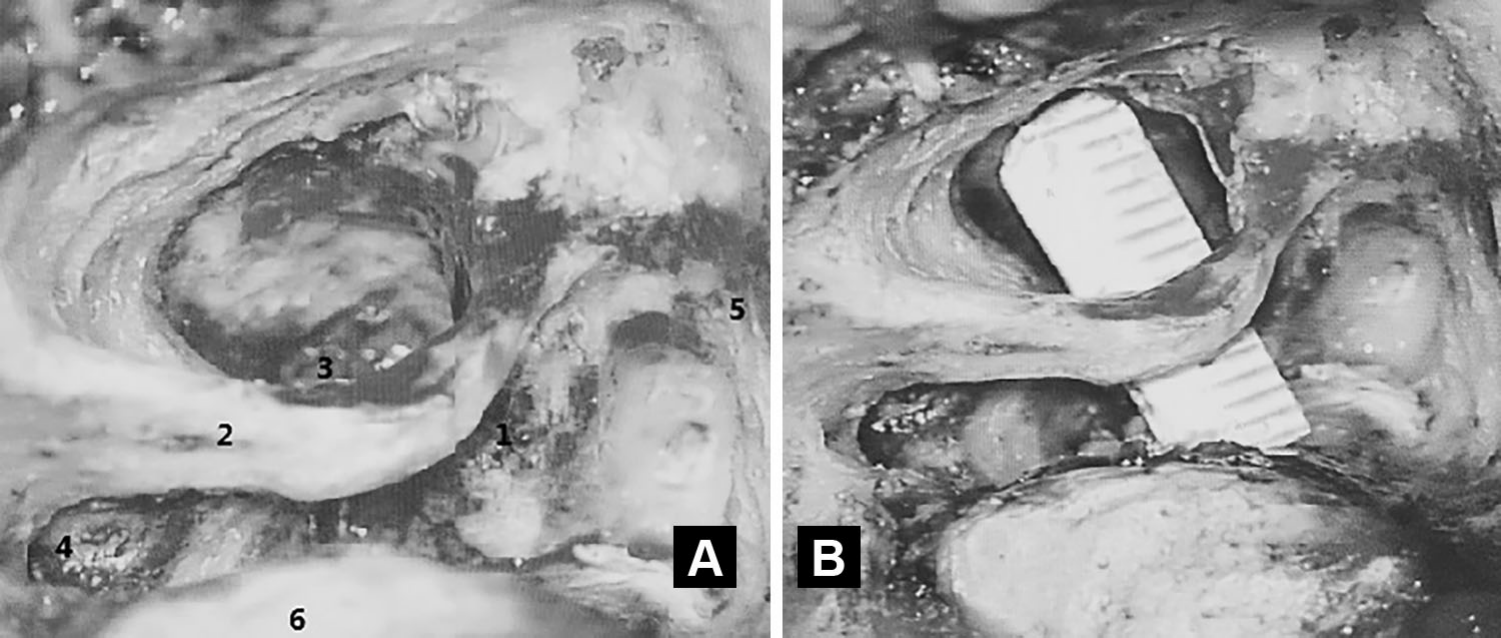
Transotic approach: intraoperative view showing the space available for exposure of the anterior cerebello-pontine angle between the fallopian canal and the vertical segment of the ICA in a temporal bone with reduced pneumatization and prominent sigmoid sinus. *1* Tumor, *2* skeletonized facial nerve, *3* internal carotid artery, *4* jugular bulb, *5* dura of middle cranial fossa, *6* sigmoid sinus

The transotic exposure continued from the subtotal petrosectomy as follows [5]. The posterior otic capsule, including the semicircular canals and posterior vestibule, was first removed to expose the posterior and inferior walls of the internal auditory canal, and then the anterior otic capsule (cochlea) was removed, drilling medial and anterior to the skeletonized fallopian canal, to expose the anterior–inferior wall of the internal auditory canal as well as the posterior fossa dura between the internal auditory canal and the exposed vertical segment of the internal carotid artery. The fallopian canal was left as a bridge across the surgical field with enough bone to prevent accidental fracture (Fig. 2). The extent of bone removal for the transotic exposure of the cerebellopontine angle gives a maximal exposure of the posterior fossa dura between the sigmoid sinus, jugular bulb, internal carotid artery and the superior petrosal sinus. A key step of the transotic approach is the exposure of the cerebellopontine angle between the skeletonized fallopian canal and ICA. This step provides exposure to the anterior part of the posterior fossa (Figs. 2, 3, 4). Only the anterior–superior wall of the internal auditory canal is left to support the intrameatal segment of the facial nerve.

Once the exposure was completed, tumor removal was begun by separating it from the intrameatal segment of the facial nerve. The posterior fossa dura was first incised between the sinodural angle and the posterior edge of the porus acusticus. The incision was then extended anteriorly below the internal acoustic porus to the level of the exposed vertical segment of the intratemporal ICA. The superior and inferior dura flaps were retracted with 4-0 Vicryl sutures, which were clipped to the drapes. The size of the tumor was reduced as much as possible by intracapsular removal with suction and biopsy forceps. Bleeders were coagulated inside the tumor with bipolar forceps. Due to the unique anterior exposure of the cerebello-pontine angle provided by the transotic approach (Figs. 2, 3, 4), it was possible to separate first the intracranial segment of the facial nerve from the anterior wall of the tumor (Fig. 3). The transotic approach also offered a perfect view of the loop of the AICA, which is usually situated along the inferior pole of the tumor. After complete separation of the tumor from the intracranial segment of the facial nerve it was possible to expose the VIII nerve for its section after coagulation of the surrounding small branches of the AICA.

Of the different techniques used for vestibular schwannoma surgery, the transotic approach offered the best conditions for a safe separation of the facial nerve from the entire anterior pole of the tumor (Fig. 3). Exposure allowing direct vision is critical because anterior to the tumor the facial nerve may be frequently spread out until it is paper thin and nearly transparent.

After tumor removal the posterior fossa dura was reconstructed with temporalis fascia or artificial dura (Fig. 2). The internal auditory canal was covered with temporalis fascia and the cavity was obliterated with fat from the lower abdominal wall wedged under the fallopian canal to compress the dura to avoid a postoperative CSF leak. At the end of surgery a temporalis muscle flap was rotated over the operative cavity and the skin incision was sutured.

## Results

1. Immediate postoperative CT scans of the temporal bones were performed 6 h after the operation to exclude intracranial hemorrhage. Facial nerve function was re-evaluated with the House–Brackmann and Fisch scale. MRI scans were taken to exclude residual tumor 3 weeks postoperatively.
2. Postoperative course: The follow-up of the patients was 1–66 months (mean 38 months), the tumor had been removed totally in 34 (94%) and near-totally in 2 patients (Table 1), The incomplete removal of two tumors was due to a firm attachment of the tumor to the brain stem; one was 4.3 cm, another was 3.2 cm. Preservation of the anatomical continuity of the facial nerve during surgery was 100% of cases (Table 2). The postoperative facial nerve function was HB grade I (resp. 100% DEFS) in 7 (19%) patients, HB grade II (resp. 81% DEFS) in 27 (75%) patients and HB grade III (resp. 59% DEFS) in 2 (6%) patients (Table 2). All patients were unilaterally deaf as before surgery (Tables 1, 2). 7 patients (19%) experienced acute postoperative vertigo, which was relieved with symptomatic treatment. There were no deaths and no severe complications such as hemiplegia, or intracranial infections; however, one patient (tumor 4.1 cm), had a postoperative intracranial hemorrhage that was resolved by reoperation, and another patient had a postoperative CSF leak that was closed by revision surgery after the failure of conservative treatment (Table 2).

## Discussion

Fisch et al. [5, 12] described the transotic approach, which Wang [13] called the “entire labyrinthine approach,” to provide better tumor exposure and facial nerve preservation than was possible with the translabyrinthine approach of W. House. Although the transotic approach may be used for all sizes of tumors, Fisch [5, 6] recommended its use for tumors up to 2.5 cm. This limitation was proposed to optimize the results by otologists performing skull base surgery, since neurosurgeons in Zürich preferred the retrosigmoid approach for the removal of larger tumors. Wang [13] proposed to use the transotic approach for tumors up to 3.5 cm. And Falcioni stated that the transotic approach was especially suitable for resection of vestibular schwannomas that involved the otic capsule [14], and Gantz et al. [15] for those that involved the vestibule and otic capsule.

The goal of the development of the transotic approach [5, 6, 14] was to provide the largest anterior transtemporal exposure of the cerebellopontine angle from the sigmoid sinus to the vertical ICA and from the superior petrosal sinus to the jugular bulb. Initially, the facial nerve was permanently transposed anteriorly [14], but very soon it was observed that the same surgical results could be obtained by leaving the fallopian canal intact and skeletonized [15].

The translabyrinthine approach provides no direct exposure of the anterior cerebello-pontine angle. With the transotic approach we were routinely able to obtain about 7 mm of exposure anteriorly to the skeletonized fallopian canal, and 4 mm posteriorly (Fig. 4). The separation of the intracranial facial nerve from the anterior pole of the tumor is done under direct vision working anteriorly to the skeletonized tympanic and mastoid segments of the fallopian canal without the need to displace the cerebellum (Figs. 3, 4).

The disadvantages of the transotic over the translabyrinthine approach are the resulting total loss of hearing and the longer operative time; on the other hand, the advantages of the transotic approach include better exposure and preservation of the facial nerve and significantly reduced incidence of immediate and delayed cerebrospinal fluid leaks with possible meningitis (Table 2) [6, 16]. Spinal fluid leaks are reduced by closure of the tympanic ostium of the Eustachian tube, obliteration of the operative cavity (subtotal petrosectomy) with abdominal fat and temporalis muscle flap, and by blind sac closure of the external auditory canal (Table 2; Fig. 2).

Our experience confirms these results (Table 2). With the transotic approach we were able to preserve the anatomic integrity of the facial nerve in all patients (Table 1). 94% of the patients had a postoperative HB grade of I or II and a Fisch DEFS of 100 or 77–83% at 6 weeks following surgery (Table 2). We believe that preservation of facial function is due to the excellent exposure given by the transotic approach for the separation of the intrameatal and intracranial segments of the nerve from the tumor (Figs. 3, 4).

Brackmann et al. [17] and Browne and Fisch [6] reported significantly fewer complications with the transotic than with the translabyrinthine approach. House [3] reported for the translabyrinthine approach the following results for postoperative facial function: 58.2% (HB I), 12.6% (HB II), 13.2% (HB III), 7.8% (HB IV), 3.3% (HB V) and 5.1% (HB VI) [3]. Fisch et al. [5] showed that the anatomic integrity of the facial nerve was preserved in 94% of the patients undergoing a transotic approach and that the functional recovery of facial function (Fisch DEFS) for tumors up to 2.5 cm was 70% (Fisch grade 100), 15% (Fisch grade 99–80), 9% (Fisch grade 79–60), 3% (Fisch grade 59–40) and 3% (Fisch grade 39–0). Our results (Table 2) for tumors up to 2.0 cm are in agreement with these results. House reported that at the time when the dura was closed with temporalis muscle (before 1974) the incidence of postoperative cerebrospinal fluid leaks for the translabyrinthine approach was as high as 20% [3], but that this rate dropped to 7% when abdominal fat was used instead of the temporalis muscle. House reported that most of the cerebrospinal fluid leaks resolved with a pressure dressing and by raising the head of the bed [7]. This indicates that the CSF leaks occurred through the surgical wound, a situation that is avoided if the abdominal fat is placed in a surgical cavity performed as a subtotal petrosectomy with occlusion of the ET and blind sack closure of the EAC. Fisch compared the incidence of postoperative CSF leaks for 147 transotic versus 114 translabyrinthine approaches [6]. Significantly fewer immediate leaks were found in the transotic (4%) than in the translabyrinthine approach (22%) in that study. The rate of delayed leaks, which can appear years after surgery and induce meningitis, was also significantly higher in cases operated with the translabyrinthine approach [6]. Our results confirm these findings (Table 2). The size of the tumor has implications for possible complications. Intracranial hemorrhage occurred in a tumor with a size of 3.8 cm and the CSF Leak in a tumor of 4.2 cm (Table 2).

Our data (Table 1) confirm that more time is needed to perform the transotic than for the translabyrinthine approach, but also that the total time of surgery is extended only a little. The longer time (2–3 h) spent developing the exposure is compensated by the more rapid removal of the tumor (1–1.5 h) (Table 1).

## Conclusions

The transotic approach has proven of value for the removal of vestibular schwannomas up to 5.0 cm in the presence of temporal bone contraction (reduced pneumatization, anteriorly located sigmoid sinus, high jugular bulb, low middle cranial fossa) as determined by preoperative imaging (CT and MRI). Hearing was not preserved, but other outcomes were favorable, including the rates of total tumor removal, preservation of facial nerve function, avoidance of CSF leaks, and severe postoperative complications.
